# Electroretinographic and Optical Coherence Tomographic Evaluations of Eyes with Vitreoretinal Lymphoma

**DOI:** 10.3390/jcm12123957

**Published:** 2023-06-09

**Authors:** Jun Makita, Yuji Yoshikawa, Junji Kanno, Yuro Igawa, Tomoyuki Kumagai, Shunichiro Takano, Takeshi Katsumoto, Takuhei Shoji, Masayuki Shibuya, Kei Shinoda

**Affiliations:** 1 Department of Ophthalmology, Saitama Medical University, Saitama 350-0495, Japan; makita.jun@gmail.com (J.M.); yujiyosi@saitama-med.ac.jp (Y.Y.); junji@saitama-med.ac.jp (J.K.); igawa@saitama-med.ac.jp (Y.I.); kaitenn314@yahoo.co.jp (T.K.); smumshun10@gmail.com (S.T.); bilstein_boxer@icloud.com (T.K.); shoojii@gmail.com (T.S.); arainko5@yahoo.co.jp (M.S.); 2 Koedo Eye Institute, Saitama 350-1123, Japan

**Keywords:** intraocular lymphoma, vitreoretinal lymphoma, electroretinogram, optical coherence tomography, vitreous biopsy

## Abstract

Vitreoretinal lymphomas (VRLs) present with different clinical characteristics. However, only a few case reports have been published that evaluated the retinal function and the retinal morphology. The relationship between retinal morphology and function of eyes with a vitreoretinal lymphoma (VRL) was investigated via optical coherence tomography (OCT) and electroretinography (ERG). The ERG and OCT findings in 11 eyes of 11 patients (69.4 ± 11.5 years old) who were diagnosed with VRL at the Saitama Medical University Hospital between December 2016 to May 2022 were studied. The decimal best-corrected visual acuity ranged from hand movements to 1.2 (median 0.2). Histopathological studies of the vitreous specimens showed class II VRL in one eye, class III VRL in seven eyes, class IV VRL in two eyes, and class V VRL in one eye. The IgH gene rearrangement was positive in three of the six eyes tested. The OCT images showed morphological abnormalities in 10 of the 11 (90.9%) eyes. Severe attenuation was found for the amplitudes of the b-wave of the DA 0.01 ERG in 6 of 11 eyes (54.5%), the DA 3.0 a-wave in 5 of 11 eyes (45.5%), the DA 3.0 b-wave in 36.4%, the LA 3.0 a-wave in 36.4%, the LA 3.0 b-wave in 18.2%, and flicker responses in 36.4% of the eyes. None of the DA 3.0 ERGs had a negative shape (b/a < 1.0). In the five eyes in which the a-wave was severely attenuated, hyperreflective dots were observed subretinally. The ERG analysis in eyes with a VRL indicates a relatively severe dysfunction of the outer retinal layer and was helpful in determining the site of the morphological changes in eyes with VRL.

## 1. Introduction

Vitreoretinal lymphomas (VRLs) are the most common intraocular lymphoproliferative disorder and are considered to be variants of primary central nervous system (CNS) lymphomas. When they occur only in the eye, they are called primary vitreoretinal lymphomas (PVRLs). A VRL can also be classified as secondary when it arises from metastasis of systemic lymphoma [1,2,3,4]. It has been reported that 13 to 25% of central nervous system malignant lymphomas are associated with intraocular lesions [5,6]. Histopathologically, VRLs consist mainly of diffuse large B-cells, and these histologic subtypes of lymphomas are called diffuse large B-cell lymphomas (DLBCL). They have a poor prognosis when accompanied by CNS lymphomas [7,8,9]. Occasionally, T-cell VRLs can also occur [10].

The most frequent ocular manifestation of a PVRL is the infiltration of lymphomatous cells into the subretinal pigment epithelium (RPE) space and the presence of single neoplastic cells in the vitreous cavity [11,12,13]. The anterior segment can also have neoplastic cells although it is less frequent than the posterior segment. In the anterior segment, it is manifested as keratic precipitates, aqueous cells, flare, and iris nodules. However, the presence of these elements are not necessary for a correct diagnosis of VRL [13,14,15].

Earlier optical coherence tomographic (OCT) studies of VRLs have shown that deposits were present above and below the RPE and infiltrations of highly reflective materials were present in the subretinal or inner retinal layers. OCT also showed a disruption of the ellipsoid zone (EZ) and clumps of cells in the vitreous cavity [16,17,18,19,20,21].

Although it is known that VRLs present with different clinical features, there are only a few case reports that evaluated the retinal function via electroretinography (ERG) [22,23,24,25,26,27] and the retinal morphology by OCT. The findings made in both systems should be helpful in cases with vitreous opacities, preventing fundus examinations. 

These limitations prompted us to conduct a study determining the morphological and functional findings in eyes with VRL via OCT and ERG, respectively. We investigated cases of vitreous opacities that led to a diagnosis of VRL from the intravitreal interleukin (IL)-10/-6 concentrations and immunoglobulin heavy chain gene (IgH) rearrangement combined with cell cytology of vitreous specimens [4,28,29,30]. We analyzed the ERGs and OCT findings acquired as close together as possible to investigate their relationships in eyes with VRL.

## 2. Patients and Methods

A review of the patients’ medical records was approved by the Ethics Committee of Saitama Medical University (IRB 2022-047). Because the study was a retrospective analysis, a waiver for informed consent was obtained. Patients who were clinically diagnosed with a VRL and had ERG recordings with the RETeval^TM^ system before the vitreous biopsy at the Saitama Medical University Hospital between 1 December 2016 to 30 May 2022 were studied. There were 11 patients (4 men and 7 women) whose ages ranged from 38 and 88 years (69.4 ± 11.5 years; mean ± SD) (Table 1). The fundus and OCT findings were extracted from the records, and the relationships between the ERG and OCT findings were determined statistically.

Prolonged steroid-resistant vitreous opacities with an average of 10.2 ± 5.8 weeks (mean ± SD) (range 2–25 wks.) were observed in all cases. A vitreous biopsy was performed during 25-gauge pars plana vitrectomy (PPV), and the vitrectomy was performed for diagnostic purposes in all cases. For the eight patients who complained of visual disturbances which was partly due to vitreous opacities, surgery was also performed for therapeutic purposes.

Pathological cytology and interleukin (IL)-6 and IL10 measurements were performed on the specimens. When the sample volume was sufficient, an immunoglobulin gene rearrangement test was performed. The presence or absence of intracranial lesions was determined via magnetic resonance imaging (MRI) examinations, and the presence or absence of systemic lesions was evaluated via positron emission tomographic (PET) examinations. 

A VRL was diagnosed in cases with high IL10/IL6 ratios and findings suggestive of VRL by gene rearrangement, or in cases with intracranial malignant lymphoma. Six cases had co-existing CNS lymphoma and five cases had primary VRL where lesions were limited to the retina and vitreous. 

Full-field ERGs were recorded using the RETeval system (LKC Technologies, Gaithersburg, MD, USA; Welch Allyn, Skaneateles Falls, New York, NY, USA), a portable ERG device that uses skin electrodes to record the retinal function. The ERGs were recorded before the vitreous surgery, and the recording conditions conformed to the standards of the International Society for Clinical Electrophysiology of Vision (ISCEV) [31]. The pupils were dilated with topical 0.5% tropicamide and 0.5% phenylephrine hydrochloride. Sensor strip skin electrodes were carefully placed 2 mm below the lower eyelid after cleaning the skin with an 80% ethanol solution and were connected to the lead wire. The ERGs were recorded under dark- and light-adapted conditions. First, after 20 min of dark adaptation, the responses to DA 0.01 and DA 3.0 were recorded; then after 10 min of light-adaptation, the LA 3.0 and flicker responses were recorded. A mini Ganzfeld dome was placed in front of the eye, and 0.01 cd·s/m^2^ and 3.0 cd·s/m^2^ flashes without background light were used to elicit the DA 0.02 and DA 3.0 ERGs, respectively, under dark-adapted conditions. Then, 3.0 cd·s/m^2^ flashes on a stable blue background light (30 cd/m^2^) were delivered with a frequency of 2.0 Hz and 28.3 Hz to elicit the LA 3.0 and flicker ERGs, respectively, under light-adapted condition. The patients were instructed to fixate on a point within the dome, and the fixation was monitored by observing the fundus with an infrared camera. 

The amplitudes of the a- and b-waves and flicker response were automatically determined using the software integrated in the RETeval^TM^ system. The ratios of the amplitudes of the a- and b-waves of the affected eye to that of the fellow eye were calculated. The ERGs were rated to 4 levels: normal with a ratio ≥ 2/3; subnormal or moderately attenuated with a ratio of 1/3 to 2/3; abnormal with a ratio < 1/3; and nonrecordable or extinguished with no response.

The OCT images were acquired with a spectral domain-OCT device (Spectralis, Heidelberg Engineering, Heidelberg, Germany) using an 8 mm horizontal and vertical cross-sectional scans centered on the fovea. In Case 11, additional scans were performed that were centered on the temporal retina corresponding to the location of the creamy subretinal lesion. The OCT findings were evaluated according to that suggested by Deák et al. [19] and Zhou et al. [20].

The patient’s age was the only continuous variable, and it is reported as the mean ± standard deviation (SD). The categorical features are reported as the count (frequency as percentages).

## 3. Results

The clinical characteristics of the patients are presented in Table 1. The BCVA ranged from hand movements to 1.2 (median 0.2, decimal BCVA). Three patients had unilateral vitreous opacities, and one patient had a phthisis in the fellow eye due to long-duration neovascular glaucoma. In the other seven patients who had vitreous opacities bilaterally, diagnostic vitrectomy was performed on eyes with more dense vitreous opacities. PPV combined with cataract surgery was performed on seven eyes with slight or mild cataracts that could affect the BCVA but not the ERG and OCT findings. Because the other two eyes were pseudophakic and two eyes had clear lens, only PPV was performed on the four eyes. Histopathological studies of the vitreous specimen showed class II VRL in two eyes, class III VRL in six eyes, class IV VRL in two eyes, and class V VRL in one eye. The IgH gene rearrangement was positive in three of the six eyes tested.

The fundus and OCT findings at 7 to 11 days before the PPV were available in eyes eyes, and vitreous opacities led to a reduction in the clarity of the fundus images in the other eight eyes. The findings in the eight eyes were obtained 1 to 13 days after the PPV. For the eight eyes, surgery was performed for therapeutic purposes. The findings in the fundus photographs and OCT images, and the electrophysiologic findings are presented in Table 2. Abnormalities were found in the OCT images of 10 (90.9%) eyes. The most frequent abnormality was irregularities of the ellipsoid zone in 6 of 11 (54.5%) eyes. Other abnormalities included focal intraretinal deposits (5 of 11, 45.5%), fuzzy outer retinas borders (5 of 11, 45.5%), hyperreflective subretinal dots (5 of 11, 45.5%), epiretinal membrane (4 of 11, 36.4%), sub-RPE deposits (4 of 11, 36.4%), preretinal deposits (3 of 11, 27.2%), subretinal fluid (3 of 11, 27.2%), retinal thickening (1 of 11, 9.1%), vertical hyperreflective lesions (1 of 11, 9.1%), and pigment epithelium detachment (PED; 1 of 11, 9.1%).

Three patients had unilateral vitreous opacities, and one patient had phthisis in the fellow eye due to a long period of neovascular glaucoma. In the other seven patients who had vitreous opacities bilaterally, diagnostic vitrectomy was performed on the eyes with more dense vitreous opacities. Fundus observations and OCT images were available, and the findings in the fellow eyes were as follows.

Multiple small lesions of chorioretinal atrophy was observed in one case, but neither inflammation nor vitreous opacities were observed. The OCT findings in the fellow eyes were symmetrical in six cases. The abnormalities included ellipsoid zone irregularities (5 of 11, 45.5%), focal intraretinal deposits (3 of 11, 27.2%), outer retina fuzzy borders (2 of 11, 18.2%), and hyperreflective subretinal dots (1 of 11; 9.1%).

The ERGs that were recorded 0 to 44 days before the PPV were analyzed. A severe attenuation or absence of the ERGs was found in the DA 0.01 b-wave of 6 of 11 eyes (54.5%), in the DA 3.0 a-wave in 5 eyes (45.5%), in the DA 3.0 b-wave in 4 eyes (36.4%), in 4 eyes (36.4%) in the LA 3.0 a-wave, in the LA 3.0 b-wave in 2 eyes (18.2%), and in the flicker responses of 4 eyes (36.4%). None of the eyes had a negative shape (b/a ratio < 1.0) for the DA 3.0 ERGs. The DA 0.01 ERGs were probably abnormal.

The a-wave tended to be equally or more severely altered than the b-wave in the DA 3.0 and LA 3.0 ERGs. In the five eyes where the a-waves (both in DA-3.0 and LA-3.0) were severely attenuated, there were hyperreflective subretinal dots present in the OCT images. In the three eyes that had normal ERGs, the ellipsoid zone was normal and hyperreflective subretinal dots were not observed.

**Figure 1:**
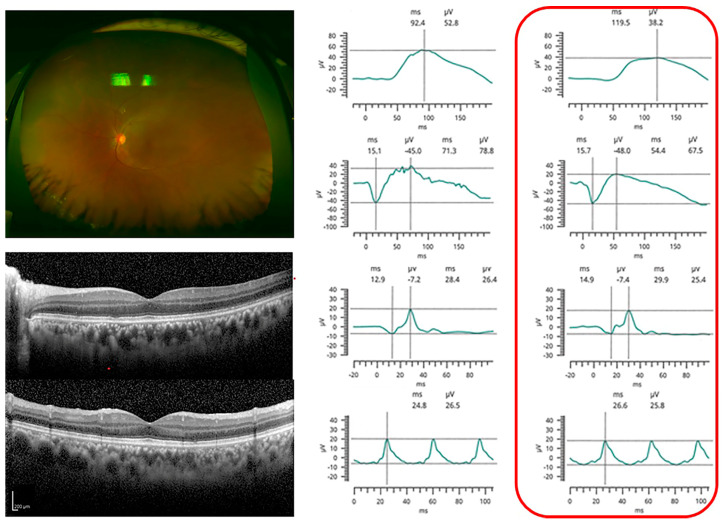
Case 3. Representative case of patient with vitreoretinal lymphoma (VRL) with normal electro-retinograms. Fundus photograph shows an area of retinal whitening in the inferotemporal periphery, which was determined as not abnormal during the surgery and afterwards for more than 4 years. The optical coherence tomographic images shows no abnormal findings. Full-field electroretinograms were normal. The red box ndicates the affcted eye.

**Figure 2:**
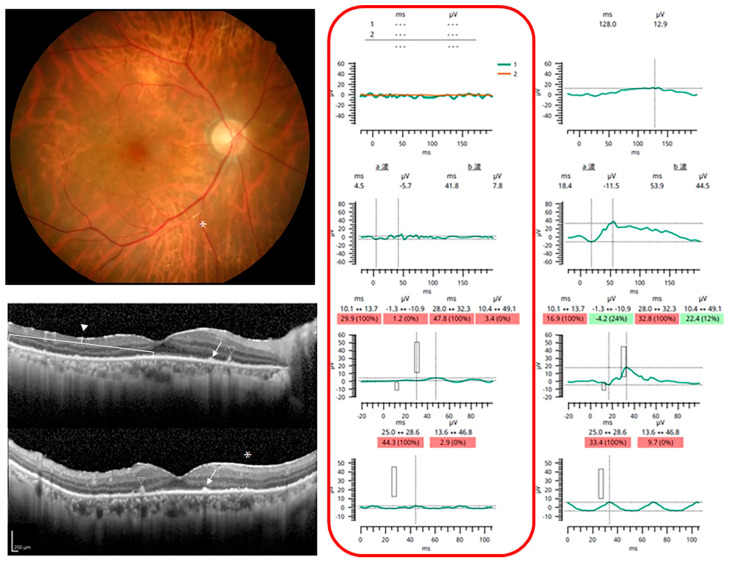
Case 5. Representative case of patient with VRL and abnormal fundus appearance and electro-retinograms. **Top left**. Fundus photograph showing a whitening of the retinal arteries (vasculitis), as indicated by asterisk (*). **Middle left** and **bottom left**: Optical coherence tomographic images showing unidentifiable ellipsoid zone, epiretinal membrane (*), focal intraretinal deposits (open triangle), fuzzy outer retinal borders (closed triangle), and hyperreflective subretinal dots (ar-rows). **Right**: Full-field electroretinograms show no response to DA 0.01 and DA 3.0, and severely attenuated response to LA 3.0 and flicker ERGs. The red box ndicates the affcted eye. a波, a wave; b波, b wave. For the implicit time, measurements from the 95th percentile to the 97.5th percentile are colored yellow and above the 97.5th are colored red. For the amplitude (and pupil area ratios), measurements from the 5th percentile to the 2.5th percentile are colored yellow and measurements smaller than the 2.5th percentile are colored red. Green coloring (or the absence of color on the device UI) is used for the remaining 95% of the range.

**Figure 3:**
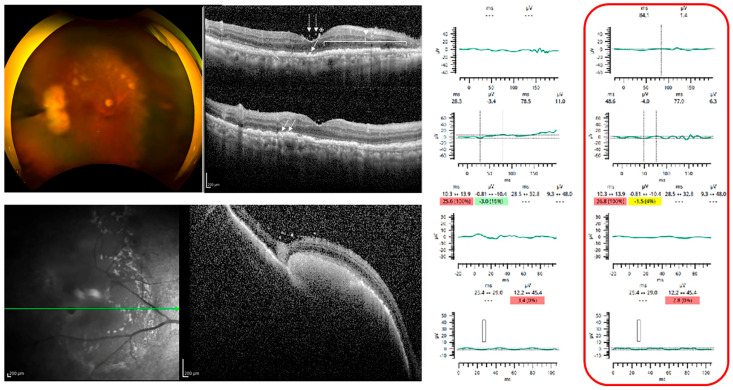
Case 11. Representative case of patient with VRL with abnormal OCT and fundus photographs and electroretinograms. **Top left**: Fundus photograph showing multiple creamy subretinal lesions (leopard spots). **Top middle**: Optical coherence tomographic (OCT) image shows unidentifiable ellipsoid zone, preretinal deposits (dotted arrow), epiretinal membrane (*), outer retinal fuzzy borders (closed triangle), and hyperreflective subretinal dots (arrows). **Bottom left**: OCT image of the area temporal to the macula shows subretinal pigment epithelium deposits (infiltration). Right: Full-field electroretinograms show no response to DA 0.01, DA 3.0, and LA 3.0, and severely attenuated flicker ERGs. The red box ndicates the affcted eye. For the implicit time, measurements from the 95th percentile to the 97.5th percentile are colored yellow and above the 97.5th are colored red. For the amplitude (and pupil area ratios), measurements from the 5th percentile to the 2.5th percentile are colored yellow and measurements smaller than the 2.5th percentile are colored red. Green coloring (or the absence of color on the device UI) is used for the remaining 95% of the range.

Representative cases are shown in Figure 1, Figure 2 and Figure 3. Case 3 had normal ERGs and no abnormalities in the fundus photographs and OCT images. Cases 5 and 11 had severe reductions in their ERG responses. Fundus examinations showed only focal arterial changes, but OCT revealed several findings in Case 5. Both the fundus and OCT examinations showed abnormalities suggestive of VRL in Case 11.

## 4. Discussion

We evaluated the retinal function using ERGs and retinal morphology using fundus photographs and OCT images in patients with VRL. The ERGs showed various changes from no responses to normal responses. Additionally, various findings were observed via ophthalmoscopy and OCT such as multiple creamy subretinal lesions (leopard spots), whitening of retinal arteries (vasculitis), preretinal deposits, epiretinal membrane, focal intraretinal deposits, outer retina fuzzy borders, and hyperreflective subretinal dots in the OCT images. 

As best we know, there has not been a study reporting a significant correlation of the ERGs with the OCT findings. However, there are several studies that focused either on the ERGs or the OCT findings. OCT and OCTA allowed for layer-by-layer assessments of the retina although in localized areas, most findings were not specific to VRL. The combined functional assessments were able to facilitate the diagnostic strength for VRLs. We are proposing functional assessments for the entire retina with layer-by-layer analysis as a utility of ERGs in VRL. 

OCT is an important and helpful noninvasive method in diagnosing and managing retinal diseases including VRL. Although several studies have been published that illustrated the OCT manifestations of primary VRLs or VRLs associated with CNS lymphomas [16,17,18,19,20,24,28,29,32,33,34,35,36,37,38,39,40,41,42,43,44,45], most of the morphologic features were nonspecific [16,32,33]. Our cases also had some of the features found in other studies; however, these features were labelled differently by different authors (Supplemental Table S1) [16,17,18,19,20,24,28,29,32,33,34,35,36,37,38,39,40,41,42,43,44,45]. It would be better if these features are named consistently in the future.

Most of the abnormalities in our cases were nonspecific to lymphomas, including epiretinal membrane, focal intraretinal deposits, outer retinal fuzzy borders, epiretinal membrane, preretinal deposits, subretinal fluid, retinal thickening, and pigment epithelial detachments. The vertical hyperreflective lesions and hyperreflective subretinal dots were considered to be associated but not exclusively with lymphomas [17,19,20].

Abnormalities were found in the OCT images in 10 of 11 eyes (90.9%) of our cohort. This incidence was within the range of previous reports, although a wide range from 37.2% to 93.8% has been reported [15,16,17,18,19,20,32,41]. The OCT findings were approximately symmetrical in both eyes, and abnormal findings were also seen in the fellow eyes. However, they were less severe, and the frequency was relatively low in the fellow eyes (6 of 11 eyes, 54.5%). This large variation in the incidence is likely due to the different inclusion criteria for patients and different scanning areas of interest. Our study included patients with an intraocular PVRL or VRL associated with a CNS lymphoma. Previous publications were review articles, retrospective case series, or case reports, therefore the scanning area by OCT varied as raster scans, only vertical or only horizontal cross-sectional scans including the fovea, and even the extramacular area. This affected the rate of detecting abnormal findings. In addition, these findings can change with time, as shown in earlier reports [17,19,20,28,41,43], and should be considered when comparing the incidence of each finding. Several authors have reported that some OCT findings such as hyperreflective subretinal change and vertical hyperreflective lesions were not consistent but seemed to be constantly evolving and resolving [19,43]. Furthermore, it is interesting to note that some changes might occur not only after treatment but also during the natural course of the disease process [19,43]. 

There are only a few studies of the ERG changes in eyes with VRL. Thus, Yasuda et al. [23] recorded ERGs in a patient with intraocular lymphoma (IOL) who had been diagnosed with a primary intracranial malignant lymphoma for three years. They reported that the PVRLs, which had a white exudate, macular retinal infiltration, and mild papillary swelling, had a negative shaped mixed rod and cone ERG response. In addition, they reported that the b-wave amplitude reduction continued even after a complete remission. Although these OCT findings are not specific features and specific correlations with the ERG findings are unclear, their case is educative and informative in that it illustrates the course of ERG findings, which was not observed in our cohort. On the other hand, Barile et al. [22] reported details of the ERG findings of a case of PVRL with progressive retinopathy. The amplitudes of the a- and b-waves of the full-field ERGs were mildly reduced with prolonged implicit times. With increasing time, there were further decreases in the amplitudes and additional 3 ms delay, while the b/a ratio was maintained at >1.0. The authors stated that the findings in their case illustrated an atypical presentation of primary IOL that was characterized by unilateral retinal disease presenting with symptoms and signs of macular dysfunction. In addition, the clinical and ERG features evolved into an acute zonal occult outer retinopathy (AZOOR)-like phenotype. Not like the abovementioned reports [22,23], the present study was performed with a cross sectional design, and no information was available for several ERG findings during the course of the disease process. Moyal et al. reported on one case of lymphoma-associated retinopathy (LAR) that was characterized by minimal morphologic changes and severe functional impairments, and the OCT images showed diffuse irregularities at the macula and distortions of the ellipsoid zone at the fovea. In addition, the a- and b-wave amplitudes were reduced [24]. The findings in their case suggested that even with minimal OCT changes, diffuse retinal dysfunction can be observed in LAR. Kim et al. presented a case of PVRL after the remission of a CSN lymphoma in which the ERGs were unrecordable in the worse eye [27]. The ERGs of the better eye were well preserved except for a marked decrease in the scotopic responses [27]. As above, eyes with VRL have different ERG findings [22,23,24,27].

In our cohort, there were no cases that had a negative-type DA 3.0 ERG, suggesting only a relatively mild functional alteration in the inner retinal layer. In the DA 3.0 and LA 3.0 ERGs, the a-wave tended to be equally or more severely altered than the b-wave. 

There were only five eyes in which both the DA 3.0 and LA 3.0 a-waves were severely attenuated, and all of these eyes had hyperreflective subretinal dots that could affect photoreceptor function (Cases 5, 7–9, and 11). This suggested a relatively severe dysfunction of the outer retinal layer. 

A reduced b-wave amplitude was observed mainly with DA 0.01. Such eyes did not always show inner retinal abnormalities in the OCT images. On the other hand, in all three eyes that had normal ERG findings, the ellipsoid zone was intact and hyperreflective subretinal dots were not observed (Cases 3, 6, and 10). The b-wave reduction may reflect a diffuse dysfunction of the inner retinal layer, and/or it may be secondary to photoreceptor damage. Our observations suggest that the outer retina was predominantly affected in our selected cases of VRL. Even in cases where vitreous opacities hampered the fundus observation, the changes in the a- and b-waves and their degree may provide information on the degree of morphological changes. 

Although we provided only descriptive data with no statistically significant correlations, we propose the importance of further investigations of the functional–morphological correlations in eyes with a VRL. The results of such studies should provide important diagnostic and prognostic information in eyes with a VRL. This is especially important because of the wide variations in the ocular characteristics of VRLs, which leads to these eyes being diagnosed with masquerade syndrome.

There are several limitations in this study. First, the number of eyes was relatively small, and this was a retrospective cross-sectional study. Recently, studies with a larger number of patients have been reported [16,17,18,20,37,41]. Most of them focused on the diagnosing methods [30,46], OCT findings [16,17,20,32], or the clinical relationship between the VRL and CNS lymphoma [3,47], and not on the ERGs. Functional evaluations with ERGs should provide clinically relevant information for understanding the pathophysiology of VRLs. Because the OCT findings can change during the natural course of VRLs, the ERGs may change in response to the treatment or even spontaneously with disease progression [23,26,48]. ERGs with skin electrodes can lessen the burden of the patients and clinic [26], repeated recordings can be considered, and longitudinal observations should obtain more detailed information of the relationship between structure and function. In addition, the OCT and ERG findings should provide information on the factors that are reversible and those that are not. 

Second, in eyes with relatively dense vitreous opacities, the OCT findings were available only after the PPV. Therefore, intravitreal and preretinal lesions may have been overlooked. Although we tried to minimize the interval between the ERG recordings and OCT imaging dates, they were not performed on the same day. This can be better accomplished with a prospective study. Third, there were no clear diagnostic criteria for VRL; in general, specimen histopathological tests are used, and the IL10/IL6 ratio and immunoglobulin H chain reconfiguration test [4,13,15,46] were comprehensively evaluated in each study group. Although the diagnosis of VRL is challenging, its methodology has been almost established, and it is necessary to increase the number of cases of VRL diagnosed with clear criteria in the future. 

In conclusion, we analyzed the ERG findings and correlated them with the OCT findings in eyes with a VRL. The functional assessments suggest a predominant impairment of the outer retinal layer and possible predictability for OCT-derived structural changes. Longitudinal studies are needed to determine the relationship between the ERG-derived retinal function and alterations in OCT-derived retinal ultrastructure.

## Figures and Tables

**Table 1:** Clinical characteristics of the patients with vitreoretinal lymphoma.

			Ophthalmic Findings					Vitreous Biopsy				sIL-2R (U/mL)	Multiple Organ Lesions
Case	Age	Gender	Laterality	Decimal VA	Best Decimal VA	Final Decimal VA	F/U Periods (mo)	Cytology, Class	IL10/IL6	IgH Gene Rearrangement	Pathological Diagnosis		
1	69	F	R	0.1	1	0.7	15	class III	undetectable	not done	DLBCL	264	Intracranial lesions
2	75	F	L	0.2	0.4	0.4	4	class III	0.72	not done	not done	627	intracranial lesions
3	38	M	L	0.5	1.2	0.7	48	class II	237.0	positive	DLBCL	not done	Intracranial lesions
4	70	F	L	0.08	0.5	0.2	11	class V	undetectable	not done	DLBCL	291	intracranial lesions
5	72	F	R	n.d.	0.04	0.04	1	class III	not done	positive	not done	190	none
6	68	F	L	0.3	1.2	0.5	30	class IV	61.8	negative	unknown	403	Intracranial lesions retroperitoneun lesions
7	64	M	R	1.2	1.2	1	42	class III	76.0	not done	not done	316	none
8	72	M	L	0.02	1.2	1.2	5	class IV	120.9	not done	not done	390	none
9	72	F	R	0.08	0.4	0.4	1	class III	31.8	negative	not done	426	none
10	75	M	L	0.3	1.2	1	7	class III	1495.4	negative	not done	248	none
11	88	F	R	n.d.	m.m.	m.m.	1	class III	3.92	positive	not done	560	none

M, male; F, female; R; right, L, left; VA, visual acuity; F/U period, follow-up period; histopathology of the vitreous specimens was performed and the Papanicolaou classification was made, if available, as class I (normal): absence of abnormal or atypical cells; class II (normal/atypical), atypical cells but no evidence of malignancy; class III (suspicious), undefined—cytology suggestive of, but not conclusive for, malignancy; class IV (suggestive), cytology strongly suggestive of malignancy; and class V (indicative), the histopathology was conclusive for malignancy; IL, interleukin; sIL2-R, soluble interleukin 2 receptor; m.m., motus manus; n.d., numerus digitorum; pathological diagnosis means diagnosis by pathologist for intracranial lesions if available; DLBCL, diffuse large B cell lymphoma.

**Table 2:** Ophthalmic findings of the patients with vitreoretinal lymphoma.

	Fundus Appearance				Optical Coherence Tomographic Findings											ERG: Amplitude (uV)						
					Entire Retina	Preretinal Lesions		Inner Layer		Outer Layer		Subretinal Lesions		RPE		Scotopc Condition				Photopic Condition		
																DA 0.01	DA 3.0			LA 3.0		Flicker
Case	Retinal Whitening around the Disc	Multiple Creamy Subretinal Lesions (Leopard Spots)	Whitening of Retinal Arteries (Vasculitis)	Chorioretinal Atrophy	Retinal Thickening	Preretinal Deposits	Epiretinal Membrane	Focal Intraretinal Deposits	Vertical Hyperreflective Lesions	Outer Retina Fuzzy Borders	Ellipsoid Zone Irregularity	Subretinal Fluid	Hyperreflective Subretinal Dots	RPE Detachment	SubRPE Deposits	b Wave	a Wave	b Wave	b/a Ratio	a Wave	b Wave	Flicker
1		+							+	+	+			+	+	↓	↓	↓	2.55	↓	↓	↓
2	+		+	+	+			+			+	+				↓↓	→	↓	1.01	↓	↓	↓
3																→	→	→	1.41	→	→	→
4			+				+	+				+				→	↓	→	3.21	→	↓	↓
5			+				+	+		+	+		+			-	-	-	n.a.	-	↓↓	↓↓
6						+										→	→	→	2.81	→	→	→
7		+		+				+		+			+		+	-	↓↓	↓	1.31	↓↓	↓	↓↓
8				+							+		+			↓↓	↓↓	↓↓	5.13	→	→	→
9		+				+		+		+	+		+		+	-	↓↓	↓↓	5.31	↓↓	↓	↓↓
10							+					+				→	→	→	1.8	→	→	→
11		+				+	+			+	+		+		+	-	-	-	n.a.	-	-	↓↓
ratio (%)	9.1	36.4	27.3	27.3	9.1	27.3	36.4	45.5	9.1	45.5	54.5	36.4	45.5	9.1	36.4	abnormal 63.6	abnormal 63.6	abnormal 63.6		abnormal 54.5	abnormal 63.6	abnormal 63.6

RPE, retinal pigment epithelium; n.a., not available. ERGs were rated to 4 levels according to the ratio of the amplitudes of the affected eye to that of the fellow eye: (→), a ratio ≥ 2/3; (↓), a ratio of 1/3 to 2/3; (↓↓), a ratio < 1/3; and (-), no response. +, positive.

## Data Availability

The data supporting the conclusions of the article are included within the article and tables.

## Supplementary Materials

The following supporting information can be downloaded at: https://www.mdpi.com/article/10.3390/jcm12123957/s1, Table S1: Various terminology for optical coherence tomographic findings in vitreoretinal lymphoma.

## Abbreviations

VRLs	vitreoretinal lymphomas
CNS	central nervous system
PVRLs	primary vitreoretinal lymphomas
DLBCL	diffuse large B-cell lymphomas
RPE	retinal pigment epithelium
OCT	optical coherence tomographic
EZ	ellipsoid zone
ERG	electroretinography
IL	intravitreal interleukin
IgH	immunoglobulin heavy chain gene
PPV	pars plana vitrectomy
MRI	magnetic resonance imaging
PET	positron emission tomographic
ISCEV	the International Society for Clinical Electrophysiology of Vision
PED	pigment epithelium detachment
IOL	intraocular lymphoma
AZOOR	acute zonal occult outer retinopathy
LAR	lymphoma-associated retinopathy

## Key Messages

1. The retinal morphology and function of eyes with a vitreoretinal lymphoma (VRL) were systematically analyzed by means of optical coherence tomography (OCT) and electroretinography (ERG), respectively.
2. Although the OCT and ERG findings were diverse and inconsistent, eyes with prominent outer retinal layer dysfunction showed outer layer abnormalities and eyes with normal ERG responses showed normal OCT findings.
3. ERG can be applied as an optimal technique that provides objective evidence for the prediction of VRL.
